# Bioremediation of coking contaminated soil: Mn(II)-oxidizing bacteria (MnOB) boost the degradation of high-molecular-weight polycyclic aromatic hydrocarbons (PAHs)

**DOI:** 10.3389/fmicb.2025.1707146

**Published:** 2025-11-21

**Authors:** Xueqin Wang, Ya-nan Wang, Yidan Yan, Yuxin Liu, Huawei Wang, Yuewei Yang, Yuanwen Liu, Yingjie Sun, Jianwei Zhao, Ying Gao

**Affiliations:** 1 School of Environmental and Municipal Engineering, Qingdao University of Technology, Qingdao, China; 2 Beijing Construction Engineering Environmental Remediation Co., Ltd., Beijing, China; 3 National Engineering Laboratory for Safety Remediation of Contaminated Sites, Beijing, China

**Keywords:** bioremediation, polycyclic aromatic hydrocarbons (PAHs), Mn(II)-oxidizing bacteria (MnOB), benzo[a]pyrene (BaP), coking soil, nitrogen stimulation

## Abstract

**Introduction:**

The Mn(II) oxidizing bacteria (MnOB) process plays an important role in persistent organic pollutant remediation, but its performance in high-molecular-weight polycyclic aromatic hydrocarbons (PAHs) degradation remains unclear.

**Methods:**

In this study, the key role of MnOB under various nitrogen conditions in enhancing the degradation of high-molecular-weight PAHs in contaminated soil after KMnO_4_ pre-oxidation was investigated.

**Results and discussion:**

The results indicated that the combination of MnOB and potassium nitrate stimulation with a C/N ratio of 100:15 was effective in degrading high-molecular-weight PAHs. 80.59% of the total PAHs and 83.77% of benzo[a]pyrene (BaP) were degraded. MnOB can effectively utilize available Mn(II) to form biogenic Mn oxides (BMOs), and the amorphous Mn oxides generated by MnOB were primarily responsible for degrading high-molecular-weight PAHs. The synergistic effect of MnOB and exogenous nitrogen was conducive to the growth and proliferation of PAH-degrading bacteria in the soil. The key functional genes involved in Mn transformation (*mntABC*), Mn oxidation (*copA, poxB, ccmC*, and *opuBD*), nitrate/nitrite reduction (*napA*, *narG*, and *nirD*), and PAH degradation (*pht3*, *pcaH*, and *pcaG*) showed strong positive correlations. The MnOB primarily drove the formation of BMOs, whose oxidative capacity was sustained via coupling with nitrate reduction, thereby enabling continuous abiotic and biotic degradation of PAHs throughout the biogenic Mn(II) oxidation process. This study provides a feasible method for promoting the remediation efficiency of high-molecular-weight PAHs in coking contaminated soil.

## 1 Introduction

Environmental pollution stemming from the coking industry has received significant global concern. Polycyclic aromatic hydrocarbons (PAHs) are representative pollutants frequently detected at contaminated coking sites (Gou et al., 2019; Zhang et al., 2021a). Due to their toxicological impacts, sixteen PAH congeners have been listed as priority control pollutants by the USEPA (Han and Currell, 2017). Among these, high-molecular-weight PAHs [i.e., benzo[a]pyrene (BaP)], which are characterized by strong hydrophobicity, poor volatility, and easy accumulation in the environment, pose a serious threat to the local environment (Li et al., 2023). Consequently, there is an urgent need to develop cost-effective remediation strategies specifically targeting high-molecular-weight PAHs in coking contaminated soils.

The primary remediation technologies for PAH-contaminated soils include washing (Bianco et al., 2022), thermal desorption (Zhao et al., 2019), chemical oxidation (Usman et al., 2018), and microbial remediation (Zou et al., 2024, 2025). Among these, chemical oxidation is the most widely applied remediation method at contaminated coking sites (Liao et al., 2019). Potassium permanganate (KMnO_4_), a strong oxidizing agent with a standard redox potential of 1.7 V, is capable of decomposing organic compounds with C = C bonds, aldehydes, and hydroxyl groups and has been frequently employed in PAH degradation (Chen et al., 2024). However, permanganate exhibits limited efficiency in oxidizing some structurally stable and recalcitrant organic contaminants, such as high-molecular-weight PAHs. During the oxidation of organic contaminants, permanganate is reduced to soluble Mn(II) ions, which may lead to secondary pollution of groundwater (Wang et al., 2023). Moreover, as a non-selective oxidant, excessive addition of permanganate can significantly reduce the soil’s organic matter, nutrient content, and microbial activity, resulting in the deterioration of soil ecological functions (Dai et al., 2022).

Microbe-mediated remediation has received widespread interest due to its mild reaction conditions, low energy requirements, and minimal risk of secondary pollution (Zeng et al., 2024a). Indigenous soil microorganisms exhibit a degree of adaptability to elevated PAH concentrations at contaminated coking sites, and the microbial populations have been shown to recover after chemical oxidation treatments (Gou et al., 2020, 2022). Nevertheless, the applicability of microbial remediation relying solely on native microorganisms is often limited by several factors: a narrow range of PAH concentrations, long remediation timescales, and unachieved remediation effect (Lü et al., 2024). For example, low- and middle-molecular-weight PAHs, such as naphthalene (36.17%), fluorene (26.96%), and benzo(a)anthracene (11.61%), can be partially degraded in contaminated soil, but biodegradation of high-molecular-weight PAHs has not been observed even after 250 days of incubation with indigenous microbes (Gou et al., 2024). To overcome these limitations, an integrated approach combining permanganate preoxidation with subsequent *in situ* biogenic stabilization mediated by Mn(II)-oxidizing bacteria (MnOB) is proposed. This approach can enhance remediation efficiency and promote the recovery of soil ecological functions.

Mn(II)-oxidizing bacteria is a class of microorganisms capable of oxidizing Mn(II) to form biogenic manganese oxides (BMOs). They can be used *in situ* to achieve adsorption immobilization of heavy metals, and degradation and mineralization of organic pollutants (Wang et al., 2020). MnOB are ubiquitously distributed in soils, sediments, aquatic systems, and even highly saline habitats. Some MnOB strains, such as *Pseudomonas putida* sp. MnB1 (Li et al., 2024), *Pseudomonas* sp. KW-2 (Wang et al., 2024), and *Comamonas* sp. RM6 (Zhao et al., 2022), have been well documented for their roles in pollutant transformation and remediation. Compared with chemically synthesized MnO_2_, BMOs exhibit lower crystallization, higher Mn oxidation states, more abundant octahedral vacancies, and larger specific surface areas (Wang et al., 2020). These structural properties endow BMOs with superior reactivity and oxidation capacity toward various pollutants such as endocrine disruptors, fungicides, and pharmaceuticals (Tran et al., 2018).

Mn(II)-oxidizing bacteria facilitates the reoxidation of dissolved Mn(II) to BMOs, thereby reducing the release of Mn(II) after KMnO_4_ oxidation and generating new reactive sites for pollutant degradation. Furthermore, the biogenic Mn(II) oxidation process produces transient reactive intermediates (i.e., superoxide and hydrogen peroxide) that significantly contribute to the transformation and degradation of organic pollutants (He et al., 2023). Despite these benefits, the process of Mn oxidation inevitably influences environmental nutrient dynamics, particularly the nitrogen cycle (Yi et al., 2022; Zhang et al., 2021b; Qiao et al., 2025). However, the potential effect of MnOB in enhancing the degradation of high-molecular-weight PAHs in contaminated coking soil with exogenous nitrogen supplementation has not yet been systematically investigated under the combined application of permanganate pre-oxidation with subsequent MnOB remediation.

Therefore, this study developed a consortium of MnOB with a high Mn(II) oxidation capacity to enhance the remediation of high-molecular-weight PAHs in coking contaminated soil following KMnO_4_ pre-oxidation. The specific objectives were to: (1) evaluate the impact of different nitrogen contents on the degradation of high-molecular-weight PAHs during the biogenic Mn(II) oxidation process; (2) clarify the dynamic changes in Mn species, bacterial community structure and metabolic function and their relationships with PAH degradation; (3) elucidate the mechanisms of Mn(II) biotransformation and PAH degradation. This research provides an integrated pre-oxidation and biostabilization strategy as a sustainable alternative for remediating high-molecular-weight PAHs in coking sites.

## 2 Materials and methods

### 2.1 Coking-contaminated soils

The coking soil samples were collected from the contaminated site of an abandoned coking plant located in Hefei city, Anhui Province, China. After removing stones, weeds, and other impurities, the soil was air-dried, homogenized, and sieved through a 60-mesh nylon sieve. The physicochemical properties of the soil samples were as follows: organic matter (OM) 82.5 g/kg, total nitrogen (TN) 4.2 g/kg, total Mn 581.2 mg/kg, and pH 8.4. The contents of the 16 priority PAHs in both raw soil and KMnO_4_-preoxidized soil are summarized in Table 1. The raw soil was mainly composed of high-molecular-weight PAHs. Among these, benzo[a]anthracene (BaA), benzo[b]fluoranthene (BbF), BaP, dibenzo[a, h]anthracene (DahA), and indeno[1,2,3-c, d]pyrene (IndP) exceeded their respective standard thresholds by 1.97, 2.89, 18.07, 3.63, and 1.23 times, respectively.

**TABLE 1:** Contents of the sixteen PAHs in the raw soil and KMnO_4_-preoxidized soil.

Compound name	Abbreviation	Rings	Standard*(mg/kg)	Raw soil (mg/kg)	Pre-oxidized soil (mg/kg)
Naphthalene	NAP	2	70	2.98 ± 0.31	N.D.
Acenaphthylene	ANY	3	/	3.53 ± 0.35	0.65 ± 0.02
Acenaphthene	ANA	3	/	2.70 ± 0.30	0.31 ± 0.01
Fluorene	FLU	3	/	2.94 ± 0.29	1.40 ± 0.04
Phenanthrene	PHE	3	/	23.74 ± 2.36	6.42 ± 0.16
Anthracene	ANT	3	/	5.03 ± 0.51	4.69 ± 0.16
Fluoranthene	FLA	4	/	40.80 ± 4.05	13.02 ± 0.43
Pyrene	PYR	4	/	23.40 ± 2.32	8.15 ± 0.24
Benzo[a]anthracene	BaA	4	15	29.49 ± 2.96	9.64 ± 0.13
Chrysene	CHR	4	1293	40.39 ± 4.03	11.22 ± 0.24
Benzo[b]fluoranthene	BbF	5	15	43.44 ± 4.35	22.92 ± 0.57
Benzo[k]fluoranthene	BkF	5	151	13.60 ± 1.36	6.81 ± 0.19
Benzo[a]pyrene	BaP	5	1.5	27.11 ± 2.68	7.42 ± 0.21
Dibenzo[a, h]anthracene	DahA	6	1.5	5.45 ± 0.55	3.05 ± 0.11
Benzo[g, h, i]pyrene	BghiP	6	/	17.11 ± 1.75	5.07 ± 0.17
Indeno[1,2,3-c, d]pyrene	IndP	6	15	18.46 ± 1.90	7.66 ± 0.14
Σ16 PAHs	/	/	/	300.15 ± 30.35	108.41 ± 6.45

*The risk screening values for soil contamination of development land class II in soil environmental quality risk control standard for soil contamination of development land (GB 36600-2018).

The purpose of KMnO_4_ pre-oxidation is to degrade a portion of PAHs, promote the decomposition of high-ring PAHs into low-ring PAHs, increase the bioavailability of PAHs, and thereby facilitate the subsequent bioremediation process. The pre-oxidation procedure was performed as follows: a 400.00 g soil sample was placed into a glass beaker and mixed with KMnO_4_ at an optimal dosage of 0.15 mmol/g dry soil (the addition amount was based on the PAH content in the dry soil). After the oxidation reaction, the soil was pH 8.5. Deionized water was added to adjust the moisture content to 30% (w/w). The soil was incubated at 25 °C and 40% relative humidity for 24 h. Following this pretreatment, the contents of residual PAHs in the soil were determined.

### 2.2 Incubation of MnOB

The complex MnOB consortium was enriched from a rust sample collected from an internal water pipe surface (Wang et al., 2024). Briefly, a 2.00 g of rust was mixed with 50 mL of sterile water and shaken at 150 rpm (30 °C) for 3 h. A 5 mL of rust-water mixture was added to 200 mL of nutrient broth (NB) medium containing 100 mg/L Mn(II) and incubated at 30 °C with shaking (150 rpm) for 5 days. The consortium underwent five domestication cycles, with each cycle lasting 5 days. Successful Mn(II) oxidation was confirmed by a color change to blue in the leucomethylene blue I (LBB) test. The MnOB cultures were harvested at the exponential growth phase for subsequent experiments, and the bacterial community structure was mainly composed of *Bacillus* (73.88%), *Pseudomonas* (14.24%), and *Clostridium* (6.56%) (Supplementary Figure 1).

### 2.3 Degradation of PAHs in pre-oxidized coking soil during the biogenic Mn(II) oxidation process

The degradation efficiency of PAHs in pre-oxidized soil was evaluated through batch experiments. The pre-oxidized soil was inoculated with MnOB and amended with varying doses of potassium nitrate to regulate C/N ratios of 100:10, 100:15, and 100:20. The experimental treatments were designed as follows: (1) MnOB addition alone (denoted as M); (2) potassium nitrate addition at three C/N levels to 100:10, 100:15 and 100:20 (denoted as N10, N15, and N20); and (3) combined MnOB and potassium nitrate additions (denoted as MN10, MN15, and MN20) (Supplementary Table 1). A treatment without the addition of MnOB and potassium nitrate was set as the control (denoted as CK). All treatments were conducted in four parallel samples.

For each treatment, 40.00 g of pre-oxidized soil samples were mixed with deionized water, MnOB (3 mL/g soil, ∼4.5 × 10^7^ cells/mL bacterial solution), and/or three doses of potassium nitrate, maintaining a solid-to-solution ratio of 1:1 (w/w). The homogenized mixture was transferred into a glass petri dish and incubated at 25 °C and 40% relative humidity. The soil sample from each treatment was collected after 7 and 14 days for analysis of PAH content and physicochemical parameters (i.e., pH, OM, and enzymatic activity).

### 2.4 Analytical methods

The content of PAHs in the soil samples was determined using high-performance liquid chromatography (HPLC). The soil samples were first extracted with dichloromethane-acetone and then monitored using an LC5090 HPLC instrument (Zhejiang Fuli Analytical Instruments Inc., China). The available Mn(II) in the soil was extracted using a DTPA solution at a ratio of 1:5 (w/v), and the concentration of Mn(II) in the solution was determined using the spectrophotometric method (Zhang et al., 2025). The free Mn oxides in the soil were extracted using a citrate-bicarbonate-dithionite procedure. Specifically, 0.50 g of soil was treated with 20 mL of 0.3 M sodium citrate and 2.5 mL of 1 M sodium bicarbonate, heated at 80 °C for 5 min, followed by addition of 0.5 g of sodium dithionite and continuous shaking for 15 min. The amorphous Mn oxides in the soil were extracted with 0.2 M ammonium oxalate by mixing 1.00 g of soil with 50 mL of extractant and shaking for 120 min. The concentrations of Mn oxides in the suspensions were analyzed using inductively coupled plasma mass spectrometry (ICP-MS) (Huang et al., 2022).

The soil samples from each treatment were subjected to microbial analysis. The total genomic DNA was extracted using a commercial soil DNA isolation kit (Qiagen, Germany). The bacterial community structure in the solid-phase waste samples was analyzed using the Illumina MiSeq 2 × 300 bp high-throughput sequencing platform at Sangon Biotech (Shanghai) Co., Ltd. The V3∼V4 region of the 16S rDNA was sequenced using the primers 341F (CCTACGGGGNGGCWGCAG) and 805R (GACTACHVGGGGTATCTAATCC).

The number of bacteria (16S rDNA gene) and *phe* gene was determined using a LightCycler 480 fluorescent quantitative PCR (qPCR) instrument (LightCycler 480 II). The primers used for amplification of 16S rDNA were 338F (ACTCCTACGGGAGGCAGCAG) and 518R (ATTACCGCGGCTGCTGG). The primers used for amplification of the phe gene were pheF (CTGCTGACSAAYCTGYTGTTC) and pheR (GGCCAGAACCAYTTRTC) (Peng et al., 2015).

### 2.5 Statistical analysis

All charts were generated using OriginPro 2025b. Pearson correlation analysis between PAH degradation efficiency and soil physicochemical properties was analyzed by SPSS Statistics 22. The soil microbial community structure was analyzed using Mothur software. Functional annotation of Mn(II) oxidation-related genes, PAH degradation genes and nitrogen metabolism genes was conducted based on the KEGG database.

## 3 Results and discussion

### 3.1 Degradation of typical PAHs during the biogenic Mn(II) oxidation process

**FIGURE 1:**
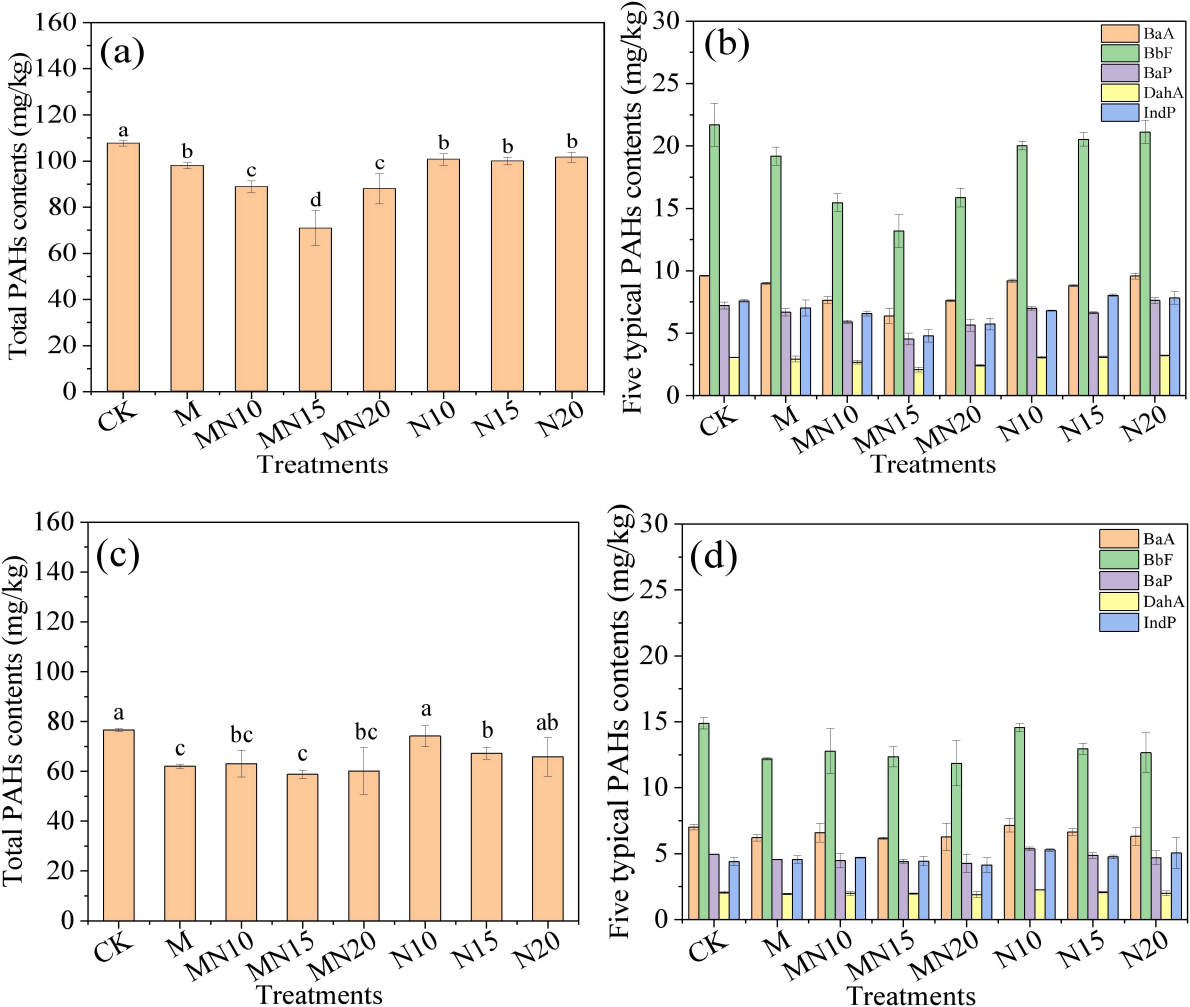
Changes in total PAHs and the contents of the five high-molecular-weight PAHs after being treated for 7 days **(a,b)** and 14 days **(c,d)**.

The changes in the contents of total PAHs and the five high-molecular-weight PAHs with different treatments are shown in Figure 1. During the KMnO_4_ pre-oxidation period, the removal efficiency of total PAHs was 63.88% after reacting for 24 h (Table 1). Similar results were reported by Gou et al. (2019), who reported a 61.7% removal of total PAHs in soil after H_2_O_2_ pre-oxidation. For the high-molecular-weight PAHs (i.e., BaA, IndP, BaP, BbF, and DahA), the removal efficiencies of BaA and IndP were 43.57% and 58.51%, respectively, and their residual contents were below the standard limit of class II in GB 36600. The removal efficiencies of BaP, BbF, and DahA were very limited, and their contents were still 4.95, 1.53 and 2.03 times higher than the standard limits of class II in GB 36600, respectively (Table 1). Therefore, the removal of these five typical high-molecular-weight PAHs by MnOB in the subsequent biological treatment was a particular focus.

During the MnOB treatment period, after 7 days of incubation, the total PAH content in the CK was 107.61 mg/kg, similar to the levels observed in KMnO_4_-pretreated CK. In the N and M treatments, the total PAH content remained near 100 mg/kg, whereas a significant reduction was observed in the MN treatments, especially in the MN15 treatment, with the lowest total PAH content of 70.76 mg/kg (Figure 1a). The MN15 treatment also exhibited the lowest residual levels for the high-molecular-weight PAHs, and the BaP content decreased from 27.11 mg/kg in the raw soil to 4.54 mg/kg (Figure 1b). After incubation for 14 days, the total PAH content in CK decreased to 78.56 mg/kg. This may be because some high-toxicity PAHs were transformed into low-toxicity PAHs after preoxidation treatment, and the native PAH-degrading bacteria in the soil gradually regained their biological activity, which could further degrade low-toxicity PAHs. The total PAH content in the MN treatments continued to decrease to approximately 60 mg/kg, which was much lower than the values in the N and CK treatments (Figure 1c). The contents of the five high-molecular-weight PAHs in the MN treatments were consistently lower than those in the CK and N treatments (Figure 1d).

These results indicated that the combination of MnOB and exogenous nitrogen during biogenic Mn(II) oxidation effectively enhanced PAHs removal in contaminated soil. In this process, MnOB biologically oxidizes Mn(II) into Mn(III/IV) oxides, which act as strong oxidants capable of directly degrading high-molecular-weight complex organic pollutants (Tran et al., 2018). Meanwhile, the addition of nitrogen not only provides essential nutrients for microbial growth (Qiao et al., 2024; Bao et al., 2020), but may also drive the oxidation of Mn(II) to Mn(IV) oxides through nitrate reduction, thereby facilitating the indirect degradation of PAHs (Cao et al., 2024). For instance, Yang et al. (2018) reported that the addition of nitrogen enhanced anoxic biodegradation of PAHs in soil. After 300 days of anoxic incubation, removal efficiencies of 3-ring and 4-ring PAHs ranged from 45% to 73% and 32%–63%, respectively. Notably, different nutrient conditions can affect the microbial metabolism of MnOB (Gonzalez and Aranda, 2023). Qi et al. (2025) suggested that under nutrient-rich conditions, multicopper oxidase predominantly drives Mn(II) oxidation, whereas under nutrient-poor conditions, the extracellular superoxide radical produced by bacteria is the main factor leading to incomplete Mn(II) oxidation. In addition, Qi et al. (2024) reported that nutrients released from *Microcystis* sp. FACHB-905 promote bacterial oxidation of Mn(II) by accelerating extracellular superoxide production.

The remediation efficacy of commonly used chemical oxidation coupled with biological enhancement on PAH degradation in contaminated soil was evaluated, with comparative results summarized in Table 2. The combined system of KMnO_4_ pre-oxidation and MnOB bioaugmentation exhibited superior performance, reducing the total PAHs content from 300.15 to 58.28 mg/kg, corresponding to a degradation efficiency of 80.59%. Moreover, the combined system shortened the remediation period from several months to 14 days. This outcome surpasses those reported in previous studies utilizing alternative combinations, such as H_2_O_2_ and indigenous microorganisms.

**TABLE 2:** Comparison of various chemical oxidation combined with microbial remediation on PAHs.

Type of soil	Method	Conditions	Initial PAHs content (mg/kg)	Treated PAHs content (mg/kg)	Efficiency	References
PAHs contaminated subsurface soil	Sodium persulfate + indigenous microorganism	10 mL 1% (wt/wt) + anoxic incubation for 80 days	142.2	≈93.18	34.47%	Gou et al., 2020
Creosote contaminated soil	O_3_ + indigenous microorganism	1.0 L/min 1.00 ± 0.02 mg/L + aerobic incubation for 56 days	2370.5	≈592.63	75%	Kulik et al., 2006
PAHs contaminated soil	H_2_O_2_ + indigenous microorganism	150 ml 10% (v/v) + anoxic incubation for 180 days	350.07	≈154.35	55.9%	Gou et al., 2019
Coking contaminated soil	KMnO_4_ + MnOB bioaugmentation	0.15 mmol/g + MnOB aerobic incubation for 14 days	300.15	58.28	80.59%	This study

### 3.2 Changes in Mn species during the biogenic Mn(II) oxidation process

**FIGURE 2:**
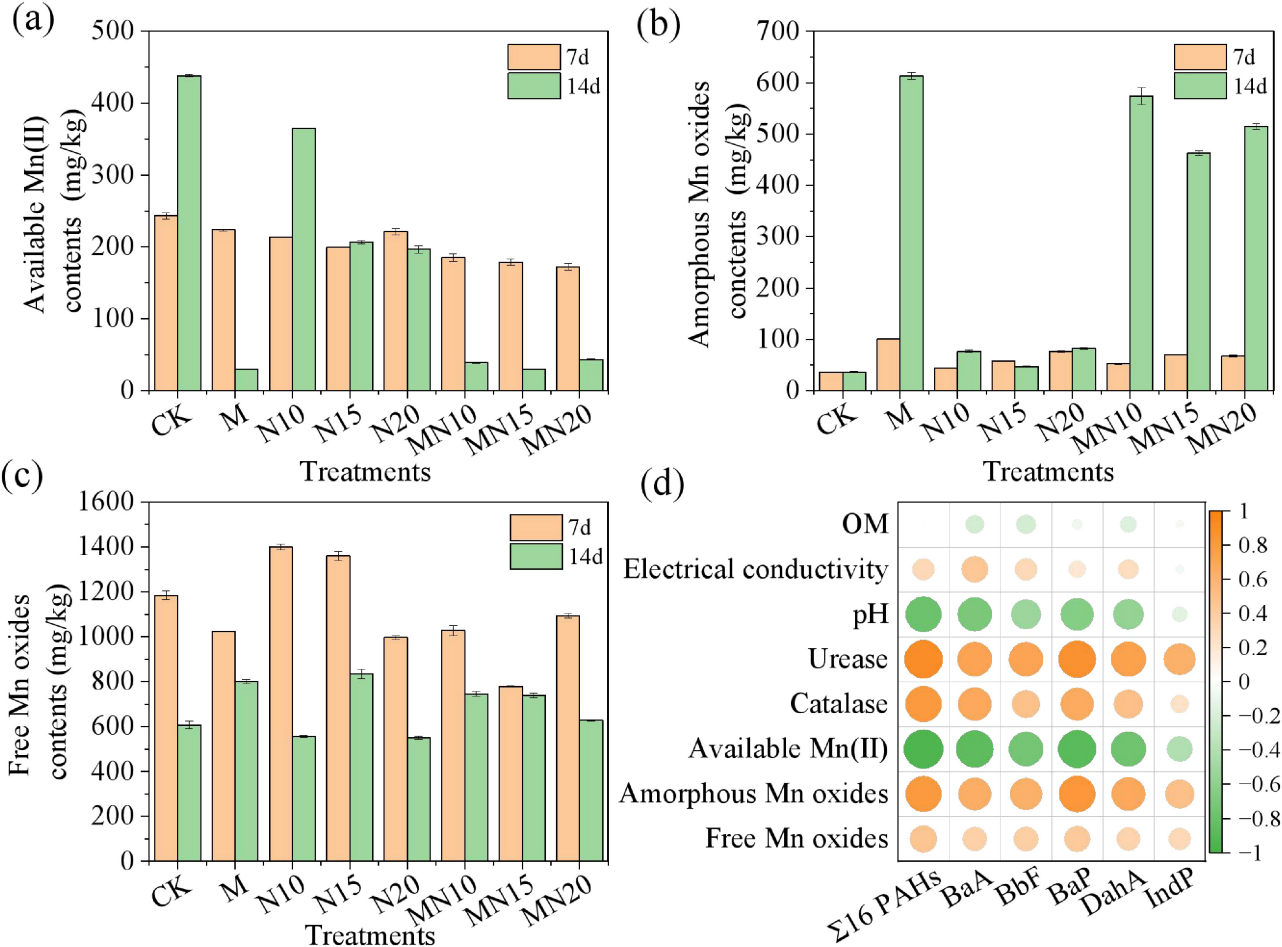
Changes in the content of available Mn(II) **(a)**, amorphous Mn oxides **(b)** and free Mn oxides **(c)**, and the relationships between the removal efficiency of Σ16 PAHs and the physical-chemical properties of soils **(d)**.

Microbial Mn(II) oxidation plays an important role in the degradation of organic pollutants (Yang et al., 2022). During this process, MnOB can convert available Mn(II) to unstable Mn(III) and then transform Mn(III) to insoluble Mn(IV), predominantly as biogenic Mn oxides (mainly BMOs) in amorphous forms. The transformation of Mn species during the biogenic Mn(II) oxidation process under different treatments is shown in Figures 2a–c. After 7 days of incubation, the contents of available Mn(II) in the MN (172.00–185.22 mg/kg) and M (223.58 mg/kg) treatments were lower than those in the CK treatment (243.22 mg/kg), whereas the content of amorphous Mn oxides in the M and MN treatments was moderately higher than that in the CK treatment. These results indicated that the available Mn(II) was transformed into amorphous Mn oxides during the biogenic Mn(II) oxidation process. After 14 days of incubation, a pronounced decrease in available Mn(II) was observed in the M and MN treatments, with values decreasing to 30.02 mg/kg in the M treatment and 29.87–43.08 mg/kg in the MN treatments, compared to 438.08 mg/kg in CK. Moreover, the content of amorphous Mn oxides increased significantly in the M and MN treatments, with values increasing from 36.14 mg/kg in the CK treatment to 612.93 mg/kg in the M treatment and 462.47–573.77 mg/kg in the MN treatments. Similarly, small increases in the contents of free Mn oxides were detected in treatments M and part of MN (MN10 and MN15), further supporting that amorphous Mn oxides were the major species of BMOs.

These results demonstrate that MnOB effectively induces the oxidation of available Mn(II) into tetravalent Mn oxides, predominantly in the form of amorphous biogenic Mn oxides. Previous studies have shown that the initial crystal morphology of microbial Mn(II) oxidation products is layered phyllomanganate Mn oxides with poor or amorphous crystallinity and primary Mn(IV) as the structural Mn (Wang et al., 2020). For example, BMO generated by *Pseudomonas putida* strain MnB1 exhibit a layered δ-MnO_2_-like structure characterized by poor crystallinity, a high average oxidation state (3.9) and a high surface area (98 m^2^/g) (Villalobos et al., 2003). Such BMOs have been widely reported to facilitate the degradation of diverse organic pollutants, including bisphenol A, phenols, atrazine, glyphosate and diclofenac (Qiao et al., 2024). It can be inferred that in the present study, the formation of amorphous Mn oxides by MnOB served as the primary contributor driving PAH degradation in the coking contaminated soils.

The Pearson correlation coefficient suggested that the degradation of PAHs was strongly negatively correlated with the available Mn(II) content and highly positively correlated with the amorphous Mn oxide content (Figure 2d). Specifically, the correlation coefficients of total PAHs and BaP with available Mn(II) were −0.96 and −0.89 (*p* < 0.01), respectively, whereas the correlation coefficients of total PAHs and BaP with amorphous Mn oxides were 0.81 and 0.84 (*p* < 0.05), respectively. These results further confirmed that biogenic Mn(II) oxidation by MnOB plays an important role in PAH degradation. Moreover, the degradation of PAHs was highly positively correlated with urease and catalase activities (Supplementary Figure 2), with correlation coefficients of 0.91 and 0.82 (*p* < 0.05), respectively. Both enzymes contributed importantly to the biogenic Mn(II) oxidation process. Andeer et al. (2015) reported that urease can increase the efficiency of Mn(II) oxidation and promote the formation of Mn oxides by adjusting the pH and nitrogen availability. Increased catalase activity could accelerate the decomposition of H_2_O_2_, thereby promoting the conversion of Mn(III) to Mn(IV).

### 3.3 Changes in bacterial number and *phe* gene absolute abundance during the biogenic Mn(II) oxidation process

**FIGURE 3:**
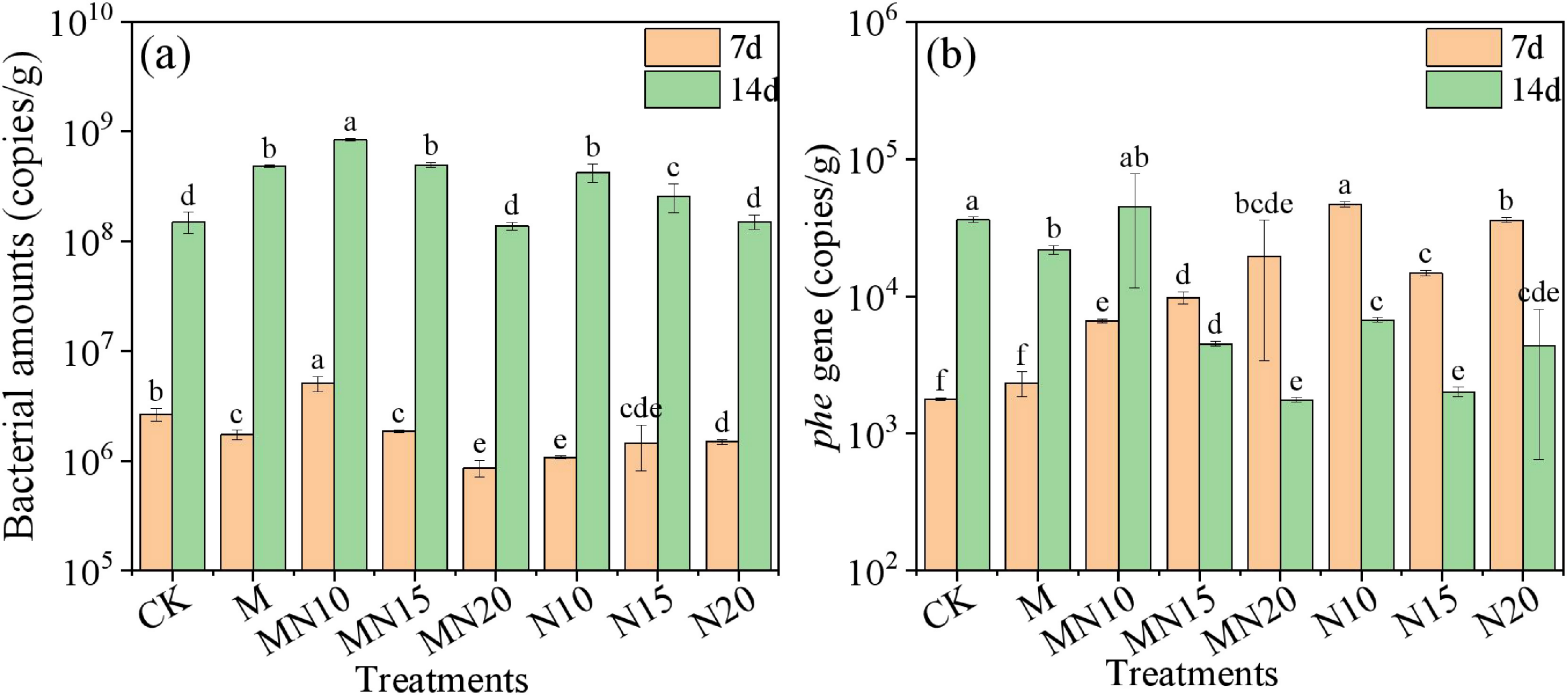
The contents of bacterial amounts **(a)** and *phe* gene **(b)** in different treatments.

The changes in bacterial number during the biogenic Mn(II) oxidation process during different treatments are shown in Figure 3a. After 7 days of incubation, the bacterial number in all treatments remained at approximately 10^6^ copies/g. The bacterial number did not significantly increase in the short period of time in the treatments with the addition of MnOB and exogenous nitrogen. This may be due to the inhibitory effect of residual KMnO_4_ in the soil. At 14 days, the number of bacteria in all treatments had significantly increased. As the inhibitory influence of KMnO_4_ diminished and highly toxic PAHs were progressively transformed into less toxic derivatives, the activity of microorganisms gradually recovered (Zhou M. et al., 2022; Zhou N. et al., 2022); therefore, the bacterial number significantly increased after 14 days of incubation. Specifically, the bacterial number in the CK treatment was 1.50 × 10^8^ copies/g, while markedly higher levels were observed in the M treatment (1.48 × 10^9^ copies/g) and moderate increases in the MN10 (8.43 × 10^8^ copies/g) and MN15 (4.97 × 10^8^ copies/g) treatments. These results indicated that the addition of exogenous MnOB combined with moderate nitrogen supplementation (C/N = 10–15) can positively enhance soil microbial proliferation. It is generally believed that the application of exogenous nitrogen by adjusting the C/N ratio from 100/10 to 100/15 enhances the activities of indigenous microorganisms and promotes the degradation of organic pollutants (Liu et al., 2021; Zhang et al., 2023).

The *phe* gene, which encodes a key enzyme involved in the metabolism of PAHs, plays an important role in catalyzing the initial hydroxylation of aromatic rings. As a rate-limiting component in PAH biodegradation pathways, it is usually used as an indicator gene for evaluating microbial PAH degradation potential (Lu et al., 2019). The absolute abundance of the *phe* gene under the different treatments is shown in Figure 3b. After 7 days of incubation, the *phe* gene abundance reached 6.64 × 10^3^–1.96 × 10^4^ copies/g in the MN treatments and 1.48 × 10^4^–4.69 × 10^4^ copies/g in the N treatments, substantially higher than that in the CK (1.78 × 10^3^ copies/g). This result indicated that the addition of exogenous nitrogen significantly promoted the activity of indigenous PAH-degrading bacteria in the soil. At 14 days, the *phe* gene abundance further increased in the M (2.18 × 10^4^ copies/g) and MN10 (4.46 × 10^4^ copies/g) treatments. In contrast, a noticeable decrease was observed in the treatments of MN15, MN20, N10, N15, and N20. This decline may be attributed to the depletion of bioavailable PAH substrates, leading to a reduction in PAH-degrading bacterial populations, particularly under elevated nitrogen conditions (Liang et al., 2022). The addition of excessive nitrogen may have also promoted the growth of other dominant bacterial communities. Although the *phe* gene abundance decreased in some treatments, BMOs present in the soil continued to decomposing PAH via abiotic oxidation (Zhou et al., 2018).

### 3.4 Changes in bacterial community structure during the biogenic Mn(II) oxidation process

**FIGURE 4:**
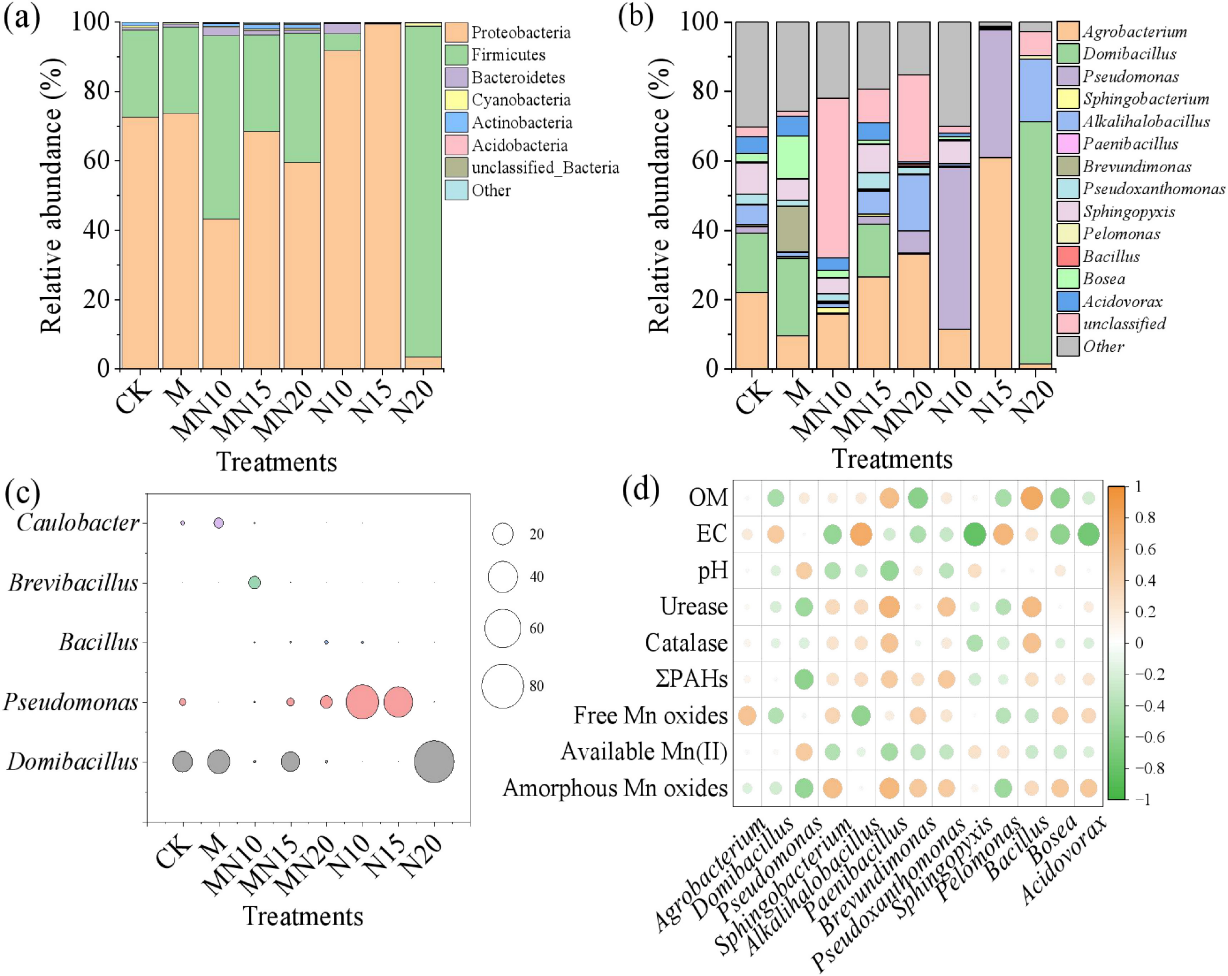
Changes in the relative abundance of bacteria at the phylum level **(a)** and genus level **(b)** in different treatments, the relative abundance of MnOB in different treatments **(c)**, and the correlation relationships between major microorganisms and environmental factors **(d)**.

The changes in bacterial structure at the phylum level after 14 days of incubation are shown in Figure 4a. Proteobacteria and Firmicutes were the dominant phyla across all treatments, consistent with their prevalence in contaminated soil environments (Phulpoto et al., 2024). Compared to the CK, the relative abundance of Firmicutes increased significantly in the treatments added with MnOB and nitrogen, reaching 52.88%, 27.88%, and 37.41% in the MN10, MN15 and MN20, respectively. The results showed that the combined addition of MnOB and exogenous nitrogen effectively enriched Firmicutes in the soil. Yuan et al. (2023) reported that the addition of nitrate increased the abundance of Firmicutes and upregulated the expression of functional genes related to PAH degradation and denitrification. Moreover, Du et al. (2022) reported that the metabolic activity of Firmicutes, such as nitrate consumption and organic matter mineralization, may be enhanced under elevated nitrogen conditions.

The changes in the bacterial microbial community at the genus level after 14 days of incubation are shown in Figure 4b. Compared to the CK, the relative abundances of *Domibacillus*, *Brevundimonas* and *Bosea* increased in the M treatment, but the abundances of *Agrobacterium* and *Sphingopyxis* decreased. In the N10 and N15 treatments, *Pseudomonas* and *Agrobacterium* were the dominant genera, with relative abundances of 46.71% and 36.89% for *Pseudomonas*, and 11.43% and 60.87% for *Agrobacterium*, respectively. In contrast, the N20 treatment was predominantly composed of *Domibacillus* (69.81%) and *Alkalihalobacillus* (18.03%). However, the diversity and composition of bacteria in the MN treatments were significantly different from those in the other treatments. *Agrobacterium* was the dominant genus in all MN treatments, with a relative abundance of 15.82%–33.16%. Meanwhile the relative abundance of *Domibacillus* greatly decreased in the MN10 and MN20 treatments, whereas unclassified genera accounted for high proportions, especially in the MN10 treatment.

The bacterial community structure of MnOB mainly consists of *Bacillus*, *Clostridium* and *Pseudomonas* (Figure 4c). However, the relative abundances of these genera in the M and MN treatments did not show a significant increase compared to the CK treatment, indicating that the introduction of MnOB did not establish a dominant bacterial community in the soils. Conversely, well-known PAH degraders, such as *Agrobacterium* and *Sphingopyxis*, were still the dominant genera in all treatments. The relative abundances of *Agrobacterium* and total bacteria in the MN15 treatment were greater than those in the CK treatment, and the removal efficiency of PAHs in MN15 was the highest, indicating that the synergistic effect of MnOB and exogenous nitrogen was conducive to the growth and proliferation of PAH-degrading bacteria in the soil. In contrast, although the M treatment had a high PAH degradation efficiency, the relative abundance of *Agrobacterium* and *phe* gene levels was lower than those in the CK treatment. This implies that the removal of PAHs was mainly mediated by the formation of BMOs during biogenic Mn(II) oxidation process rather than by MnOB bacteria.

The correlations between major microorganisms and environmental factors are shown in Figure 4d. Amorphous Mn oxides were positively corrected with *Paenibacillus*, *Pseudoxanthomonas*, *Bacillus* and *Sphingobacterium*. The free Mn oxides were corrected with *Agrobacterium* and *Sphingobacterium*. *Pseudoxanthomonas* and *Agrobacterium* have been shown to contribute to the degradation of PAHs in soil (Kuppusamy et al., 2016). *Bacillus* has been found to eliminate 99% of phenanthrene, benzo[a]fluoranthene and pyrene from contaminated coking soil (Nan et al., 2022). Overall, the addition of nutrients and MnOB can not only increase the degradation of PAHs, but also alter the structure of the soil microbial community.

### 3.5 Changes in bacterial metabolic function during the biogenic Mn(II) oxidation process

Based on the KEGG pathway analysis at level 3, the relative abundance of pathways related to PAH degradation, the nitrogen cycle and Mn oxidation is analyzed (Supplementary Figures 3a–c). Inoculation with MnOB and/or nitrogen source led to a notable elevation in the abundance of key metabolic pathways, including ABC transporters, PAH degradation, benzoate degradation, lipopolysaccharide biosynthesis, glycolysis/gluconeogenesis, drug metabolism-cytochrome P450, alanine, aspartate, and glutamate metabolism, and two-component system. The correlation analysis revealed significant relationships between the two-component system and ABC transporters with multiple metabolic processes, such as PAH degradation, fluorobenzoate degradation, benzoate degradation, lipopolysaccharide biosynthesis, butanoate metabolism, glycolysis/gluconeogenesis, and pyruvate metabolism (Supplementary Figure 3d). ABC transporters play a critical role in maintaining the homeostasis of essential transition metals such as Mn and iron, as well as in regulating the active uptake of nitrate (Li et al., 2024). The two-component system is a central signaling pathway in bacteria that regulates various physiological processes including growth, stress response, and environmental adaptation.

Benzoate degradation serves as a key downstream pathway in the metabolic breakdown of various PAHs, such as phenanthrene, pyrene, and fluorene, making it one of the dominant routes within the heterologous biodegradation network. Pyruvate metabolism in glycolysis/gluconeogenesis yields energy and substrates for the tricarboxylic acid (TCA) cycle or fermentation, producing essential precursors like ATP and pyruvate. The addition of both MnOB and nitrogen significantly increased the abundance of the alanine, aspartate, and glutamate metabolic pathways. These pathways provide essential intermediates to support microbial energy metabolism (Tang et al., 2025). Moreover, the relative abundance of cytochrome P450 in MN15 exceeded that in both M and CK treatments. Cytochrome P450 monooxygenase catalyzes the initial oxidation of PAHs to catechol, which is further cleaved by catechol dioxygenase and eventually enters the microbial TCA cycle (Silva et al., 2023).

Overall, these metabolic enhancements indicated that the combined application of MnOB and nitrogen effectively stimulates PAH degradation and supports primary metabolic pathways (such as energy metabolism), reinforcing the effectiveness of the combined remediation strategy.

### 3.6 Changes in genes related to biogenic Mn(II) oxidation and PAH degradation

**FIGURE 5:**
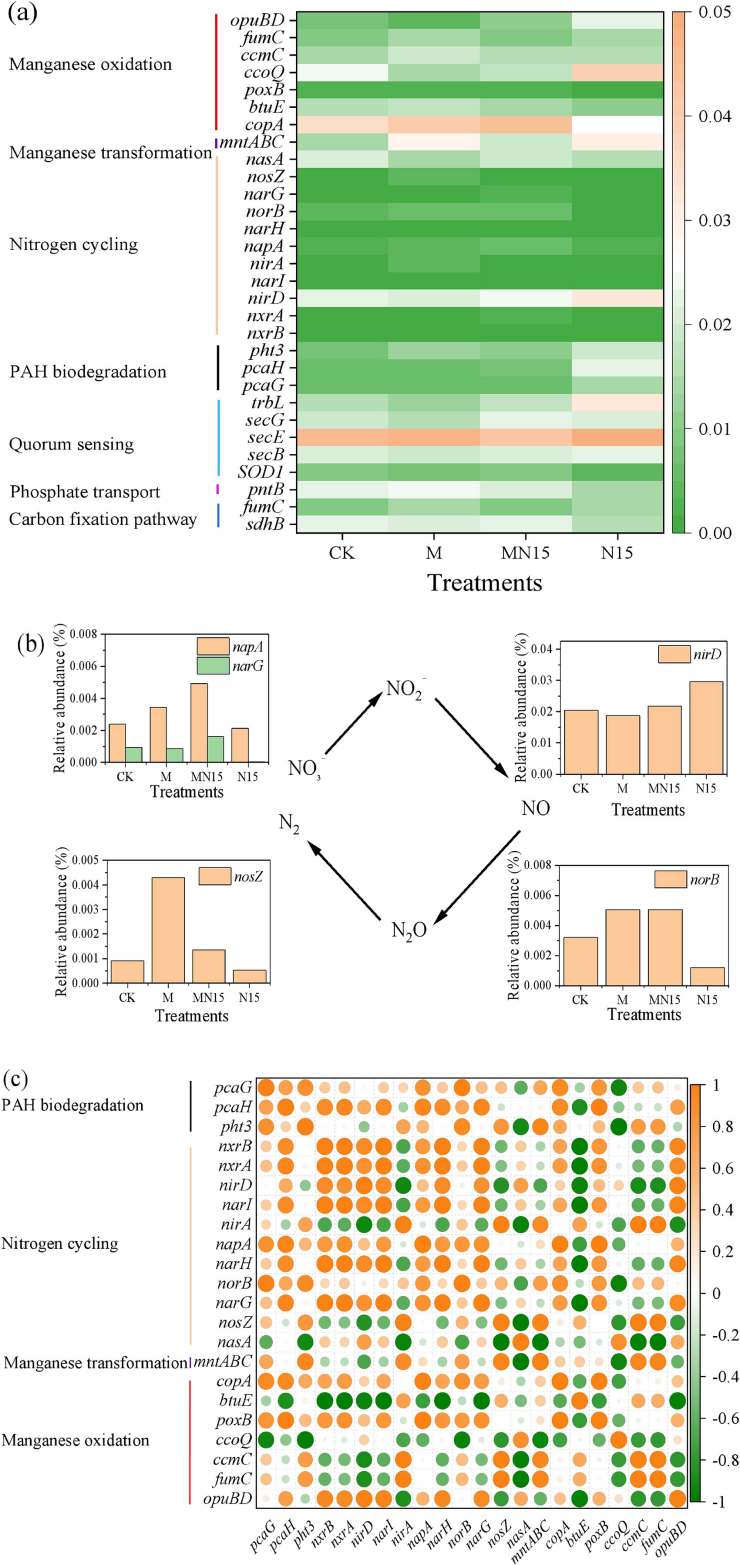
Relative abundances of the genes related to Mn(II) oxidation, the nitrogen cycle, and PAH degradation **(a)**, the relative abundance of nitrogen reduction genes **(b)**, and the correlation analysis of key genes associated with Mn oxidation, nitrogen cycling, and PAH degradation **(c)**.

Based on the gene function annotation from KEGG, the key genes related to Mn(II) oxidation, the nitrogen cycle, PAH degradation, and cellular metabolism were identified (Figure 5a). The addition of MnOB and nitrogen led to a notable increase in the abundance of the *mntABC* gene cluster. The *mntA*, *mntB*, and *mntC* genes encode the ATP-binding protein, the permease protein, and the substrate-binding protein of the Mn transport system, respectively. This upregulation implies an enhanced Mn uptake capacity, potentially supporting a more efficient cellular metabolism linked to Mn utilization. Compared to CK, the addition of MnOB and nitrogen increased the abundance of PAH degradation genes, such as *pht3*, *pcaG*, and *pcaH* genes. This was in accordance with the degradation efficiency of PAHs in these treatments. In the M treatment, the abundances of the *nirA*, *napA*, *norB* and *nosZ* genes were slightly higher than those in the CK. In the MN15 treatment, the abundances of the *norB* and *napA* genes were significantly higher than those in the CK. The elevated levels of *napA* in both the M and MN15 treatments suggest enhanced nitrate reduction to nitrite following MnOB inoculation (Figure 5b).

Correlation analysis further revealed significant associations among the key functional genes involved in Mn transformation (*mntABC*), Mn oxidation (*copA*, *poxB*, *ccmC*, *opuBD*), nitrate/nitrite reduction (*napA*, *narG*, *nirD*), and PAH degradation (*pht3*, *pcaG*, *pcaH*) (Figure 5c). Specifically, Mn transformation genes and most Mn oxidation genes showed strong positive correlations with both PAH degradation genes and nitrate reduction genes. The gene relationship supports the presence of a coupled metabolic network in which Mn oxidation is linked to PAH degradation and nitrate reduction. Zeng et al. (2024b) reported that a significant coupling between nitrate reduction and Mn oxidation was observed in strain MFQ7, where Mn(II) acted as an electron donor to enhance denitrification. Furthermore, the observed coordination between nitrate/nitrite reduction genes and PAH degrading genes aligns with the study by Jin et al. (2024), which found a positive correlation between the abundance of electron transfer chain genes (such as *narGHI*, *napAB*, etc.) and PAH degradation genes, reinforcing the metabolic synergy underlying the co-remediation process observed in this study.

### 3.7 Degradation mechanism of PAHs during the biogenic Mn(II) oxidation process

**FIGURE 6:**
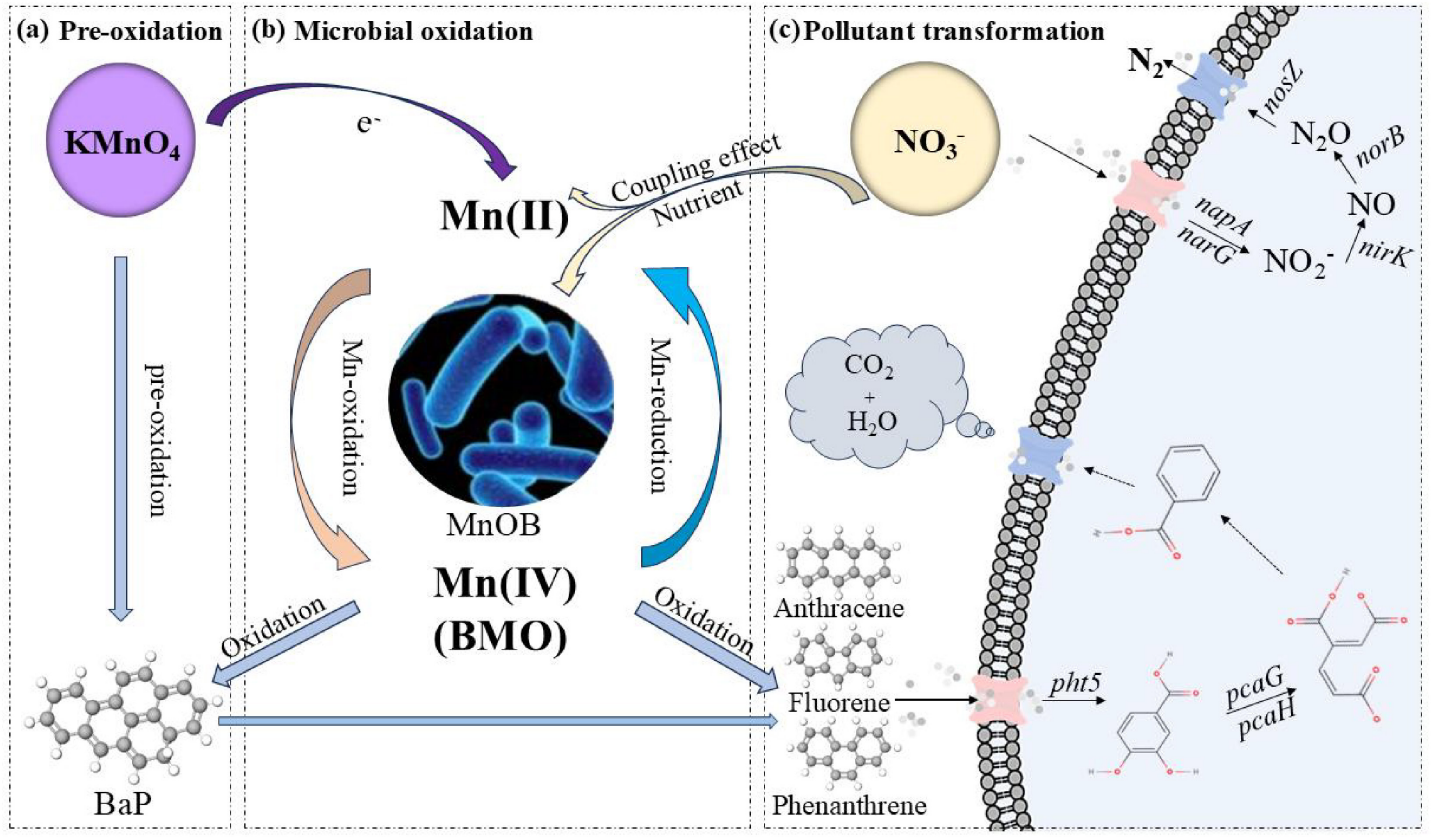
Schematic diagram of potential mechanism for PAH degradation during the biogenic Mn(II) oxidation process. **(a)** Pre-oxidation. **(b)** Microbial oxidation. **(c)** Pollutant transformation.

Based on the above results, the degradation mechanism of PAHs during the biogenic Mn(II) oxidation process is inferred, as illustrated in Figure 6. The main process mainly included KMnO_4_ pre-oxidation, Mn(II) oxidation to BMOs driven by MnOB, and the degradation of high-molecular-weight PAHs. The nitrogen cycle (driven by nitrate reduction) provided a more favorable nutritional environment and energy flow for the entire microbial community, and formed a coupling effect of nitrate reduction with biogenic Mn oxidation. KMnO_4_ pre-oxidation partially degrades BaP (a representative high-molecular-weight PAH), generating intermediates such as PHE and benzo [a] pyrene 4,5-dione. Meanwhile, Mn(VII) is reduced to soluble Mn(II) during the degradation of BaP. This newly available Mn(II) subsequently serves as a key substrate for MnOB.

Mn(II)-oxidizing bacteria adsorbs a large amount of available Mn(II) onto the surface of bacterial cells and oxidize it into Mn(III) through enzymatic reactions (e.g., multicopper oxidase). Then Mn(III) is further oxidized into Mn(IV) through a disproportionation reaction (Andeer et al., 2015; Learman et al., 2011). Under anoxic environment, nitrate can serve as an electron acceptor for microbial-driven Mn (II) oxidation, thereby facilitating the oxidation of Mn(II) to Mn(IV) (Cao et al., 2015). During the biogenic Mn(II) oxidation process, BaP is oxidized to benzo [a] pyrene 4,5-dione, which is further transformed into 1,2-dihydroxyanthraquinone, and ultimately mineralized to CO_2_ and H_2_O (Wang et al., 2022). The Mn(II) regenerated during BMO-mediated BaP oxidation can be reutilized by MnOB, establishing a sustainable redox cycle and continuously generating reactive surfaces for pollutant degradation (Wang et al., 2024). In addition, highly active intermediates such as ⋅O_2_^–^ and H_2_O_2_ produced during Mn(II) oxidation may also participate in the degradation of BaP (Qi et al., 2024). During the subsequent biological treatment period, the low-toxicity PAH intermediates are further degraded by PAH-degrading microorganisms expressing key functional genes such as *pht3*, *pcaG*, and *pcaH*.

Overall, KMnO_4_ pre-oxidation reduced the content and toxicity of PAHs, providing available Mn(II) for MnOB. The MnOB then induced the formation of BMOs, whose oxidative capacity is sustained via coupling with nitrate reduction, thereby enabling continuous abiotic and biotic degradation of PAHs throughout the biogenic Mn(II) oxidation process.

## 4 Conclusion

The combined system of KMnO_4_ pre-oxidation and MnOB bioaugmentation exhibited superior performance in reducing the total PAHs content in coking-contaminated soil. KMnO_4_ pre-oxidation reduced the content and toxicity of PAHs, providing available Mn(II) for MnOB, MnOB can effectively utilize available Mn(II) to form BMOs, which are primarily responsible for the degradation of high-molecular-weight PAHs. Inoculation with MnOB and nitrogen source had a high removal capacity for total PAHs and BaP, and the abundance of key metabolic pathways was also notably elevated. The key functional genes involved in Mn transformation, Mn oxidation, nitrate/nitrite reduction, and PAH degradation showed strong positive correlations. The MnOB primarily drove the formation of BMOs, whose oxidative capacity was sustained via coupling with nitrate reduction, thereby enabling continuous abiotic and biotic degradation of PAHs throughout the biogenic Mn(II) oxidation process.

## Data Availability

The data presented in the study are deposited in the figshare repository under DOI 10.6084/m9.figshare.30549821. Further enquiries regarding the raw data presented in the study are to be directed to the corresponding author.
