# Supplementary material for: Bioremediation of coking contaminated soil: Mn(II)-oxidizing bacteria (MnOB) boost the degradation of high-molecular-weight polycyclic aromatic hydrocarbons (PAHs)
Source: Front Microbiol. 2025 Nov 21;16:1707146. doi: 10.3389/fmicb.2025.1707146 (PMC12679158; doi:10.3389/fmicb.2025.1707146)
Supplement: Supplementary file 1 [file Data_Sheet_1.pdf]

Supplementary materials

Title

Bioremediation of coking contaminated soil: Mn(II)-oxidizing bacteria (MnOB) boost the degradation of high-molecular-weight polycyclic aromatic hydrocarbons (PAHs)

Authors

Xueqin Wang[a1], Ya-nan Wang[a1], Yidan Yan[a], Yuxin Liu[a], Huawei Wang[a*], Yuewei Yang[b,c], Yuanwen Liu[b,c], Yingjie Sun[a], Jianwei Zhao[a], Ying Gao[a]

Affiliations

a. School of Environmental and Municipal Engineering, Qingdao University of Technology, Qingdao 266520, China;

b. Beijing Construction Engineering Environmental Remediation Co., Ltd., Beijing 100015, China;

c. National Engineering Laboratory for Safety Remediation of Contaminated Sites, Beijing 100015, China.

[1] These authors contributed equally to the work.

Corresponding author

Dr. Huawei Wang: wanghuawei@qut.edu.cn; wanghuawei210@163.com

Table S1 The experimental design

Items		Treatment
CK	No N and MnOB	/
N10	Nitrate only	0.1688 g KNO_3（C/N=100:10）
N15	Nitrate only	0.859 g KNO_3（C/N=100:15）
N20	Nitrate only	1.549 g KNO_3（C/N=100:20）
M	MnOB only	3 ml/g cells
MN10	MnOB+Nitrate	3 ml/g cells+0.1688 g KNO_3（C/N=100:10）
MN15	MnOB+Nitrate	3 ml/g cells+0.859 g KNO_3（C/N=100:15）
MN20	MnOB+Nitrate	3 ml/g cells+1.549 g KNO_3（C/N=100:20）

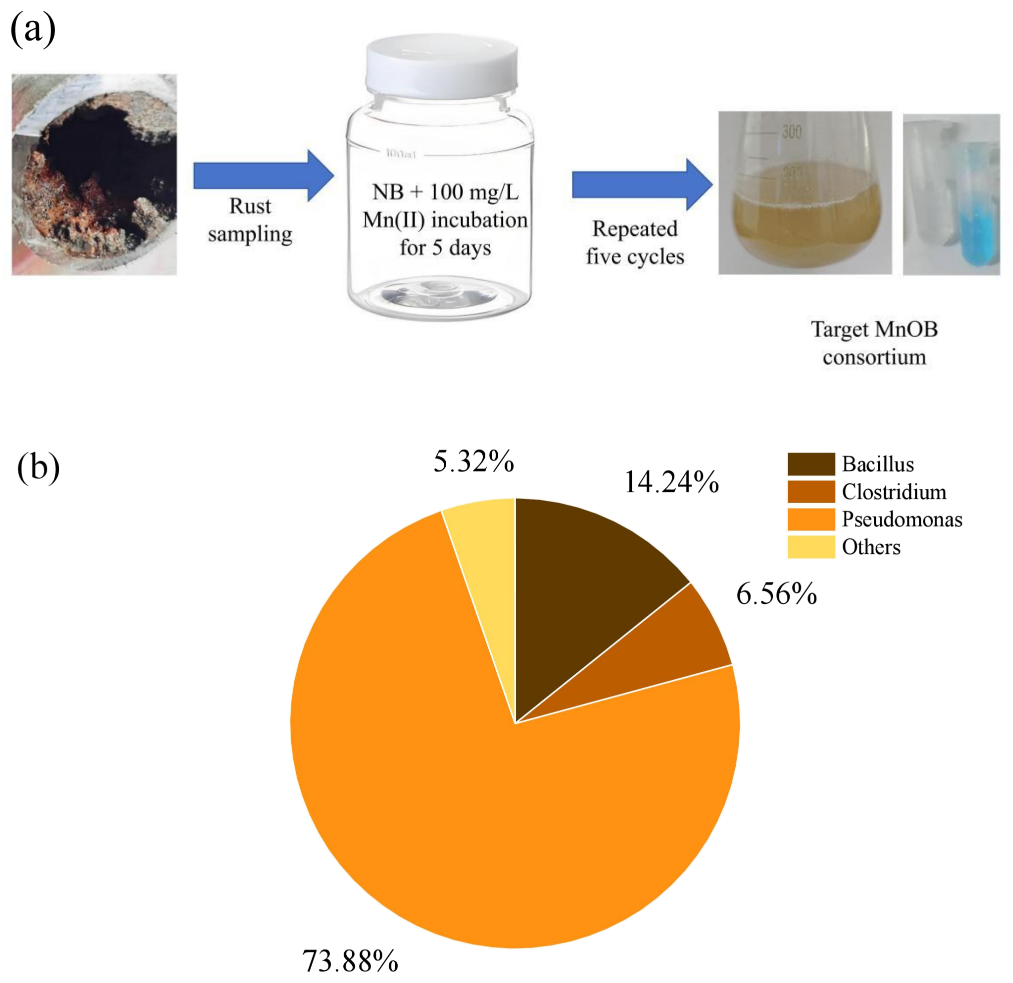

Fig. S1 The flow of obtained MnOB consortium (a) and the main composition of MnOB (b).

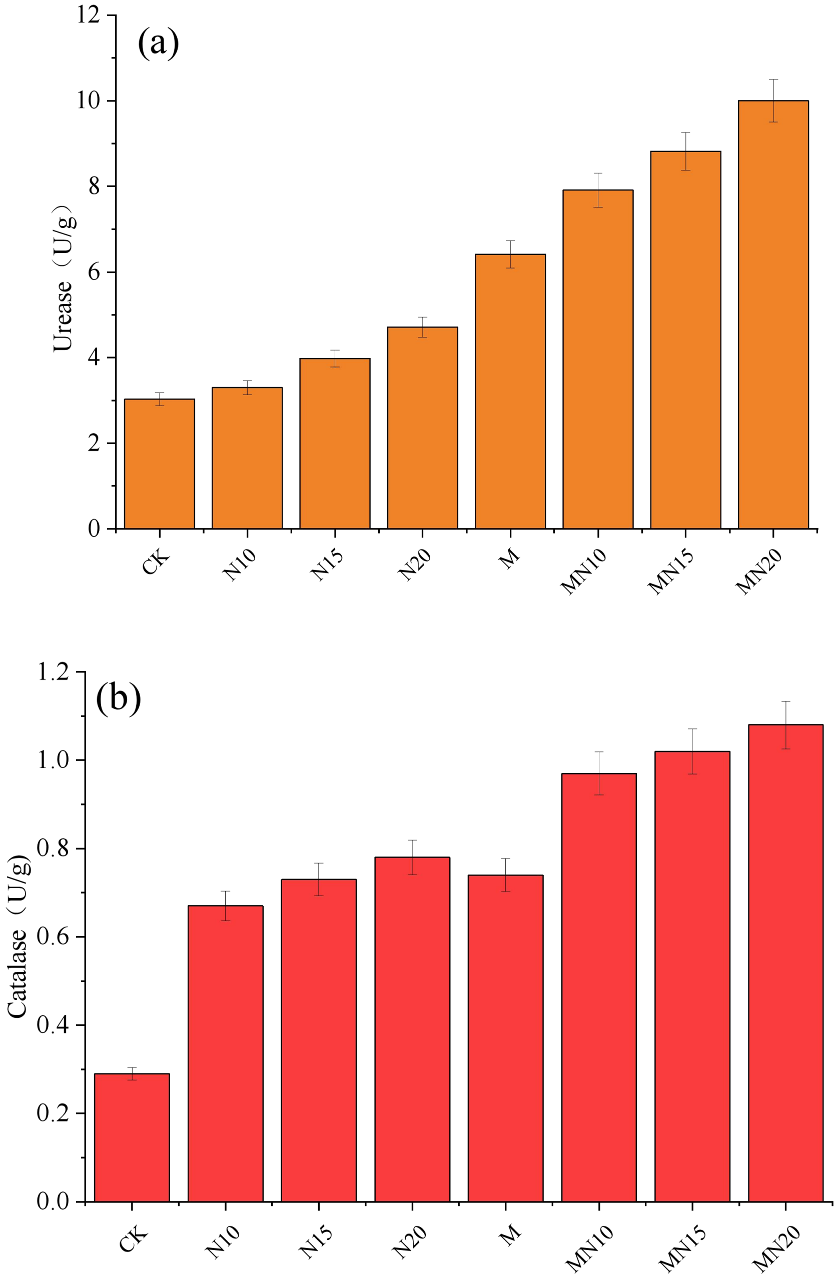

Fig. S2 Changes of urease (a) and catalase (b) activities with different treatments.

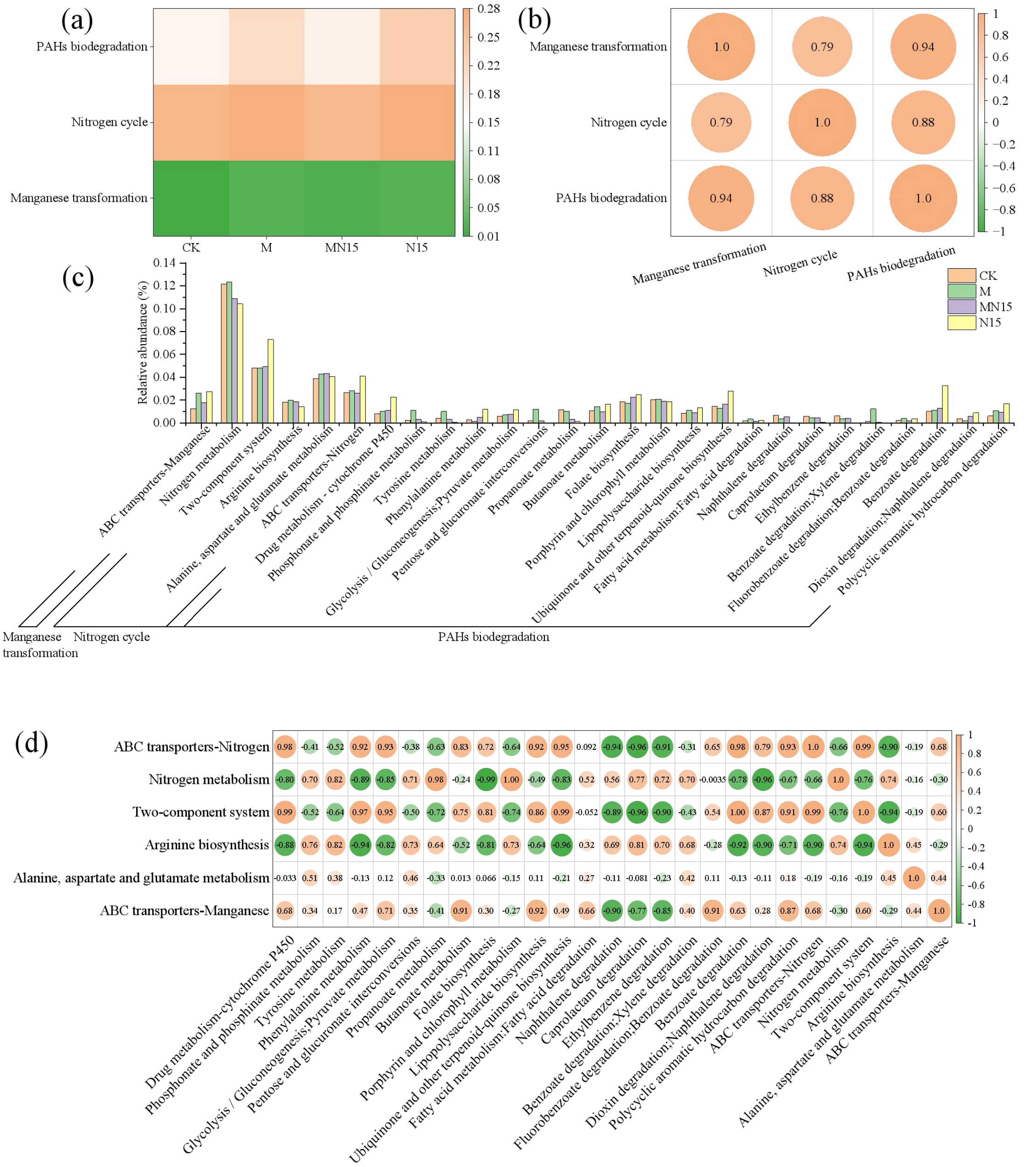

Fig. S3 Relative abundance of the bacterial metabolic function (a), the correlation analysis of major metabolic pathways (b), the relative abundance of major metabolic pathways involved in PAHs degradation, manganese transformation and nitrogen cycle in the KEGG categories (at level 3) (c), and the correlation analysis of major metabolic pathways involved in PAHs degradation, Manganese transformation and nitrogen cycle (d).
